# Sports-related sudden cardiac deaths in the young population of Switzerland

**DOI:** 10.1371/journal.pone.0174434

**Published:** 2017-03-28

**Authors:** Babken Asatryan, Cristina Vital, Christoph Kellerhals, Argelia Medeiros-Domingo, Christoph Gräni, Lukas D. Trachsel, Christian M. Schmied, Ardan M. Saguner, Prisca Eser, David Herzig, Stephan Bolliger, Katarzyna Michaud, Matthias Wilhelm

**Affiliations:** 1 University Clinic for Cardiology, Inselspital, Bern University Hospital, University of Bern, Bern, Switzerland; 2 University Heart Center, Zurich, Switzerland; 3 Institute of Forensic Medicine, University of Zurich, Zurich, Switzerland; 4 Unit of Forensic Medicine, University Center for Legal Medicine, Lausanne, Switzerland; University of Miami School of Medicine, UNITED STATES

## Abstract

**Background:**

In Switzerland, ECG screening was first recommended for national squad athletes in 1998. Since 2001 it has become mandatory in selected high-risk professional sports. Its impact on the rates of sports-related sudden cardiac death (SCD) is unknown.

**Objective:**

We aimed to study the incidence, causes and time trends of sports-related SCD in comparison to SCD unrelated to exercise in Switzerland.

**Methods:**

We reviewed all forensic reports of SCDs of the German-speaking region of Switzerland in the age group of 10 to 39 years, occurring between 1999 and 2010. Cases were classified into three categories based on whether or not deaths were associated with sports: no sports (NONE), recreational sports (REC), and competitive sports (COMP).

**Results:**

Over the 12-year study period, 349 SCD cases were recorded (mean age 30±7 years, 76.5% male); 297 cases were categorized as NONE, 31 as REC, and 21 as COMP. Incidences of SCD per 100,000 person-years [mean (95% CI)] were the lowest in REC [0.43 (0.35–0.56)], followed by COMP [1.19 (0.89–1.60)] and NONE [2.46 (2.27–2.66)]. In all three categories, coronary artery disease (CAD) with or without acute myocardial infarction (MI) was the most common cause of SCD. Three professional athletes were identified in COMP category which all had SCD due to acute MI. There were no time trends, neither in overall, nor in cause-specific incidences of SCD.

**Conclusions:**

The incidence of SCD in young individuals in Switzerland is low, both related and unrelated to sports. In regions, like Switzerland, where CAD is the leading cause of SCD associated with competitions, screening for cardiovascular risk factors in addition to the current PPS recommendations might be indicated to improve detection of silent CAD and further decrease the incidence of SCD.

## Introduction

Sudden cardiac death (SCD) in the young and apparently healthy individuals is a devastating event with enormous impact on community health [1]. Reduction of cardiovascular mortality is expected due to growing increase in awareness of risk factors over the past few decades, but the SCD burden worldwide is still huge [2]. Despite numerous reports on the topic, our knowledge about the precise incidence, demographics, causes and circumstances surrounding the issue remain limited, as do regional differences [3].

SCD is a leading cause of death in competitive athletes [4]. The crucial role of regular physical activities in reduction of mortality from cardiovascular disease is widely known and is documented by several studies [5, 6]. However, strenuous physical activities may provoke life-threatening arrhythmias in certain individuals at-risk [7]. Therefore, preventive measures are required to protect susceptible individuals from exercise-related fatalities.

Aiming to reduce the rates of sports-related SCD, considerable importance has been given to pre-participation screening (PPS) for early detection of silent cardiovascular pathologies that predispose athletes to effort-induced life-threatening conditions. However, investigators are still in search for more advanced screening methods that would detect the majority, if not all individuals with silent, potentially fatal heart disease [8, 9]

In Switzerland, PPS including an ECG has become mandatory for athletes in certain high risk sports (e.g. ice hockey, soccer) since the end of 2001. The remaining athletic population is screened on a voluntary basis and only 9% of non-elite athletes are screened [10]. The impact of ECG-based PPS on rates of SCD related to exercise is unknown.

In Switzerland, unexpected deaths are reported to the district attorney, who then initiates an inquiry to determine the manner of death (natural, accidental, homicidal or suicidal). The preliminary forensic examination is an investigation of the scene, evaluation of the medical history and thorough external examination of the corpse, mostly performed by a forensic pathologist. The district attorney decides if further examination, such as an autopsy, is necessary in a case-based approach [11]. Based on a recent study in the Swiss canton of Vaud, the autopsy rate in young SCD victims is 47.5% [12]. This autopsy rate seems valid for both sports-related SCDs and SCDs unrelated to exercise, and applicable to all cantons [11].

We aimed to analyse the total, as well as sex-, and cause-specific incidences and time trends of sports-related SCD in comparison to SCD unrelated to exercise in Switzerland.

## Methods

### Study population

We retrospectively reviewed all forensic autopsy reports of the German-speaking part of Switzerland (population in the examined region is 5,617,963, which comprises nearly 70% of the Swiss population) for sudden unexpected deaths in individuals aged 10–39 years, occurring between 1999 and 2010. Deaths were classified as SCD when they occurred within 24-hours of symptom onset or beginning of physical activity, and either autopsy had identified a cardiac pathology as the probable cause of death; or no obvious causes had been identified by the post-mortem examination and therefore a fatal arrhythmia was the likely cause of the event.

In Switzerland, forensic autopsy reports contain extensive information on autopsy results and circumstances of death, which are stored in case-record databases of the Forensic Medical Institutes. For efficient determination of manner and etiology of SCD, information on timing of symptoms and activity preceding the event are obtained from bystanders, family members and/or other witnesses, and are recorded in the forensic autopsy report.

Cases of SCD were classified into three categories based on whether or not sports were performed within the 24-hours preceding the SCD: no sports (NONE), recreational sports (REC), or competitive sports (COMP). Sports-related SCDs were included in COMP if they were related to official athletic competitions, intended as an organized team or individual sport event, placing high premium on athletic excellence and achievement [8]. All other exercise-related SCDs were categorized as REC. The timing of SCD was related to the onset of symptoms (NONE) or the physical activity (REC, COM), classified into three groups: instantaneous, within one hour, and within 24-hours [3].

Examination of the heart was performed by local forensic pathologists, and the post-mortem diagnosis was based on macroscopic findings. When needed, microscopic and toxicological examinations were performed to clarify the underlying pathology. The criterion for hemodynamically relevant CAD was a lumen narrowing of ≥50% [12]. Data were recorded in anonymized and standardized fashion in the electronic SWISS REGistry of Athletic Related Death (www.swissregard.ch) [13].

### Data analysis

The population data were derived from the Swiss Federal Statistical Office and a survey on sports-participation in Switzerland from the Swiss Federal Office of Sports. The Swiss Federal office of Statistics provided yearly population data of residents aged 10–39 years from the German speaking cantons [14]. The survey on sports participation provided extensive data on sporting activities, as well as volume and intensity of training [15]. A core representative sample of more than 10,000 15- to 75-year-old residents was first questioned by telephone, followed by an online questionnaire on their sports participation. Children aged 10 to 14 years were assessed with specially adapted questionnaires. The survey provided the ratios of people engaged in recreational or competitive sports of the concerned age group. Based on this, denominators for our three categories were formed as follows: all residents aged 10–39 years (denominator for NONE category); subjects engaged in sports (denominator for REC); and participants of competitions (denominator for COMP). Average yearly incidences were calculated for the pre-screening (1999–2001), early screening (2002–2004), mid-screening (2005–2007) and late screening (2008–2010) periods.

Data distribution was assessed by Shapiro-Wilk test. Continuous variables were compared with the Student’s *t*-test or Mann-Whitney *U* test, as appropriate. Categorical data were analysed with Chi-squared test or Fisher´s exact test in case of low field numbers. *P*-values of all outcomes were two-sided and a value less than 0.05 was considered significant.

Since our cohort included only autopsied SCD cases, we multiplied the recorded numbers by a factor of 2.1 to adjust the numbers of SCDs for the average autopsy rate of 47.5% in Switzerland in order to estimate real incidences [11, 12].

The data was analysed with SPSS statistical package (SPSS Software for Windows, v. 17.0) for descriptive statistics. Time trends were calculated by Poisson log-linear regression analysis, and average yearly incidences for pre-, early-, mid-, and late screening periods were calculated per 100,000 person-years (statistical software R, R Core Team, 2015). For SCD rates, 95% confidence intervals were calculated based on Poisson distribution with exact methods using the software package epiR.

### Ethics

This study was evaluated by the Ethics Committee of the canton of Bern, Switzerland; for analysis of health-related personal data no ethical approval was required. According to the Swiss Law on Human Research, informed consent from next-of-kin for the retrospective analysis of anonymised data was not necessary.

## Results

### Characteristics of victims and the events

Over the observed period, the average annual population aged 10–39 years in the studied region was 2,112,038 persons, with similar number of male (50.8%) and female inhabitants [14]. Around 73% of men and women were engaged in sports, of whom 20% were involved in competitions [15]. The number of residents, their sports participation rate, age and sex distribution remained stable over the observed period [14, 15].

Overall, 82 women (23.5%) and 267 men (76.5%) aged 10–39 years suffered SCD in the German-speaking part of Switzerland, accounting for an overall incidence of 2.89/100,000 person-years (mean age ± SD, 30±7 years; 76.5% male). Among all 349 individuals, 297 (85.1%) deceased without an obvious connection to physical exercise (NONE), 31 cases (8.9%) were related to recreational sports (REC) and 21 (6%) to competitive sports (COMP). Incidences of SCD [mean (95% CI)] were the lowest in REC [0.43 (0.35–0.56)], followed by COMP [1.19 (0.89–1.60)] and the highest in NONE [2.46 (2.27–2.66)]. The incidence of SCD unrelated to sports (NONE) was nearly 4 times higher compared to sports-related SCD (2.46 vs. 0.59).

We noted predominance of male victims in all three categories, and no significant difference in mean age of subjects between groups (p = 0.732). The proportions of males in REC (96.8%) and in COMP (85.7%) were greater than in NONE (73.7%) (p = 0.009). Out of 52 sports-related deaths, 48 occurred in men (92.3%). Three professional male athletes were included in COMP, aged 26, 28 and 30 years.

The majority of SCDs occurred instantaneously or within 1 hour after the onset of initial symptoms or sports. This tendency was the highest in REC (87.1%), and higher in COMP (85.7%) than in NONE (53.9%). We observed the highest rate of witnessed cases in COMP (85.7%), followed by REC (64.5%) and NONE (42.7%) (Table 1).

**Table 1:** Circumstances of sudden cardiac deaths and additional examinations performed at autopsy.

	No sports	Recreational	Competitive	*P*
	(NONE)	sports (REC)	sports (COMP)	
	N = 297	N = 31	N = 21	
Characteristics of the events				
Timing of SCD				<0.001
instantaneous	100 (33.7%)	12 (38.7%)	12 (57.1%)	
within one hour	60 (20.2%)	15 (48.4)	6 (28.6%)	
within 24 hours	137 (46.1%)	4 (12.9%)	3 (14.3%)	
Witnessed	127 (42.7%)	20 (64.5%)	18 (85.7)	<0.001
Resuscitation performed	181 (60.9%)	28 (90.3%)	18 (85.7)	<0.001
Additional examinations performed at autopsy				
Microscopic autopsy (n)	260 (87.5%)	28 (90.3%)	19 (90.5%)	0.844
Toxicology (n)	205 (69.0%)	23 (71.8%)	16 (76.2%)	0.717
Body mass index (kg/m^2^)	24.7±6.3	24.0±4.7	25.5±3.8	0.680
Heart weight (g)	420±143	411±103	448±136	0.633

COMP, SCD related to competitions; NONE, SCD not related to physical activities; REC, SCD associated with physical activities other than competitions; SCD, sudden cardiac death

### Distribution of underlying causes

**Fig 1:**
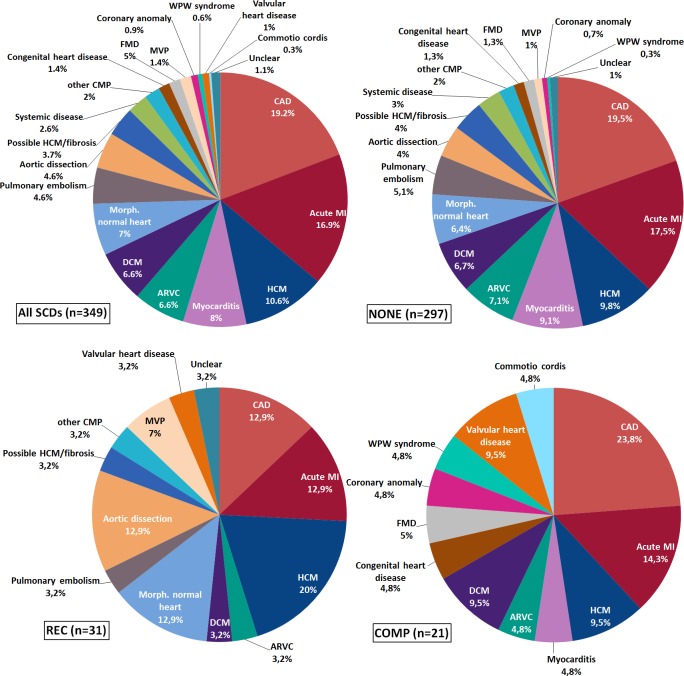
Distribution of underlying causes of sudden cardiac death and their relation to exercise. ARVC, arrhythmogenic right ventricular cardiomyopathy; CAD, coronary artery disease; CMP, cardiomyopathy; COMP, SCD related to competitions; DCM, dilated cardiomyopathy; FMD, fibrous muscular dysplasia of coronary artery; HCM, hypertrophic cardiomyopathy; MI, myocardial infarction; MVP, mitral valve prolapse; NONE, SCD not related to physical activities; REC, SCD associated with physical activities other than competitions.

CAD with or without acute myocardial infarction (MI) was the most common causative pathology in all three groups; cause-specific incidences were 0.91 (0.80–1.03), 0.11 (0.07–0.18), and 0.45 (0.28–0.73) for NONE, REC and COMP, respectively (Fig 1). Overall, premature CAD accounted for death of 126 young individuals (89% male; 52% ≤35 years), comprising 36% of all SCDs.

Amongst all sports-related SCDs, 13.4% were due to acute MI, all cases being observed below 35 years of age. In young male competitive athletes (age≤35 years) acute MI was the most common cause of SCD. Premature CAD with or without MI was responsible for 8 deaths in COMP, which all occurred instantaneously or within 1-hour of exertion. All females who died from acute MI (7 cases) were aged >25 years, and none of these deaths were related to sports, while four young men aged ≤25 years died from acute MI.

In young women, aged ≤35years, cardiomyopathies were the leading pathological substrate for SCD (40.7% of cases). In older females, CAD was more commonly observed (42%). Death of three women was related to participation in competitive sports (COMP); they succumbed to death due to WPW syndrome, myocarditis and valvular heart disease. The only female victim in REC category had a morphologically normal heart on autopsy.

Among cardiomyopathies, hypertrophic cardiomyopathy (HCM) was the most frequent; incidences in NONE, REC and COMP were 0.24 (0.19–0.31), 0.09 (0.05–0.15) and 0.11 (0.05–0.29), respectively. In around half of SCD victims with underlying HCM, death was related to recreational sports. There were 13 additional cases (12 in NONE and 1 in REC) where autopsy revealed myocardial fibrosis and (borderline) left-ventricular hypertrophy, which could possibly have been HCM; these were classified in a separate group (possible HCM/fibrosis) due to unfulfilled histologic criteria (myocardial disarray) for post-mortem definite diagnosis of HCM.

**Fig 2:**
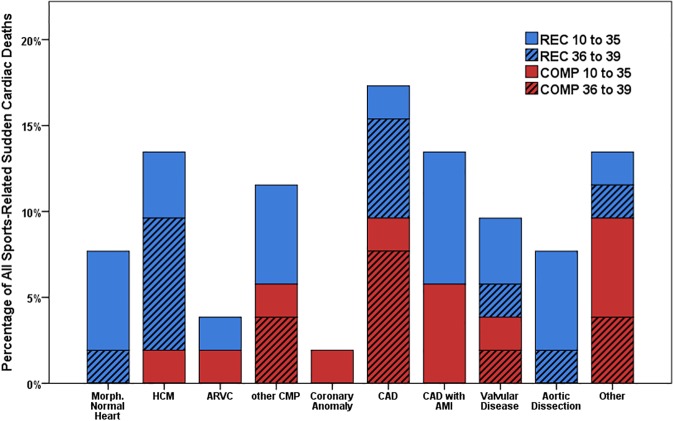
Distribution of underlying causes of sports-related sudden cardiac death. Distribution of underlying causes of sudden cardiac death (SCD) and their relation to recreational (REC) and competitive sports (COMP) in age groups of 10–35 and 36–39 years. AMI, acute myocardial infarction; ARVC, arrhythmogenic right ventricular cardiomyopathy; CAD, coronary artery disease; CMP, cardiomyopathies; COMP, SCD related to competitions; DCM, dilated cardiomyopathy; HCM, hypertrophic cardiomyopathy; REC, SCD associated with physical activities other than competitions; SCD, sudden cardiac death.

**Fig 3:**
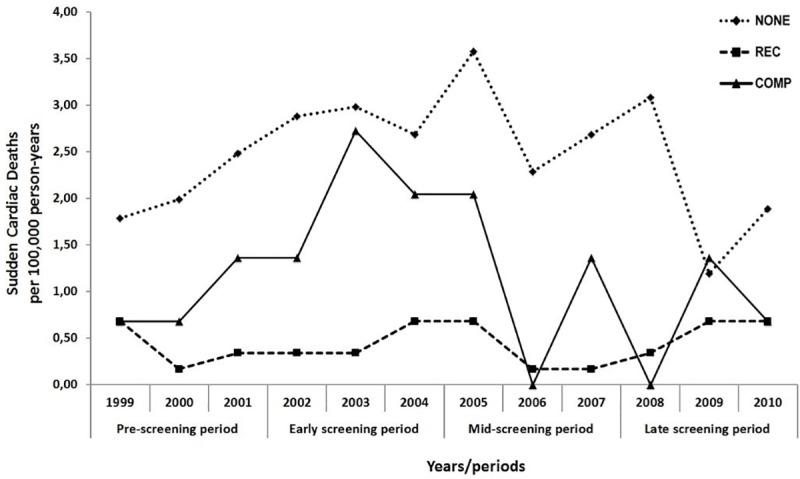
Annual incidence rates of sudden cardiac death in the young population of the German-speaking region of Switzerland. Young population included individuals aged 10 to 39 years. Sudden cardiac deaths (SCDs) occurring between 1999 and 2010 were evaluated. Incidence rates are presented for NONE, REC and COMP categories classified based on relation of the SCD to sports. COMP, SCD related to competitions; NONE, SCD not related to physical activities; REC, SCD associated with physical activities other than competitions.

Sports-related SCDs from other underlying cardiomyopathies were rare, only 13% of sports-related deaths were due to dilated cardiomyopathy (DCM), arrhythmogenic right ventricular cardiomyopathy (ARVC) or any other cardiomyopathy (Fig 2). ARVC was considered the causative pathology in 2 cases, one in REC and one in COMP. All victims of ARVC and coronary artery anomalies were aged 35 years or younger. Two of the three professional athletes died during wrestling and one during ice hockey. No time trend for total or cause-specific incidences of SCD was found in any of the groups (Fig 3 and Table 2).

**Table 2:** Numbers and annual incidence rates and trends of total and cause-specific sudden cardiovascular deaths, based on their relation to sports in relation to 3-year periods.

			Periods								
			Pre-screening period		Early screening period		Mid-screening period		Late screening period		
			(1999–2001)		(2002–2004)		(2005–2007)		(2008–2010)		
		Total No. of events	No. of events	Incidence rate (95% CI)	No. of events	Incidence rate (95% CI)	No. of events	Incidence rate (95% CI)	No. of events	Incidence rate (95% CI)	*P* ^c^
**Total No. of events in NONE**		297	63	2.09 (1.76–2.48)	86	2.85 (2.46–3.30)	86	2.85 (2.46–3.30)	62	2.05 (1.73–2.44)	0.912
CAD		110	24	0.80 (0.60–1.05)	32	1.06 (0.84–1.35)	36	1.19 (0.95–1.50)	18	0.60 (0.43–0.82)	0.814
	MI	52	13	0.43 (0.30–0.63)	11	0.36 (0.24–0.55)	18	0.60 (0.43–0.82)	10	0.33 (0.22–0.51)	0.949
Cardiomyopathies		82	19	0.63 (046–0.86)	24	0.80 (0.60–1.05)	26	0.86 (0.66–1.12)	13	0.43 (0.30–0.63)	0.762
	HCM	29	4	0.13 (0.07–0.26)	7	0.23 (0.14–0.38)	12	0.40 (0.27–0.59)	6	0.20 (0.12–0.34)	0.792
	Possible HCM/ fibrosis^a^	12	3	0.10 (0.05–0.21)	3	0.10 (0.05–0.21)	3	0.10 (0.05–0.21)	3	0.10 (0.05–0.21)	0.916
	ARVC	21	3	0.10 (0.05–0.21)	8	0.26 (0.17–0.43)	6	0.20 (0.12–0.34)	4	0.13 (0.07–0.26)	0.976
	DCM	20	9	0.30 (0.19–0.47)	6	0.20 (0.12–0.34)	5	0.17 (0.09–0.30)	0	0	0.430
Myocarditis		27	8	0.26 (0.17–0.43)	9	0.30 (0.19–0.47)	5	0.17 (0.09–0.30)	5	0.17 (0.09–0.30)	0.693
Morphological normal heart		19	4	0.13 (0.07–0.26)	5	0.17 (0.09–0.30)	4	0.13 (0.07–0.26)	6	0.20 (0.12–0.34)	0.908
Pulmonary embolism		15	1	0.03 (0.01–0.12)	5	0.17 (0.09–0.30)	3	0.10 (0.05–0.21)	6	0.20 (0.12–0.34)	0.602
Aortic dissection		12	4	0.13 (0.07–0.26)	4	0.13 (0.07–0.26)	3	0.10 (0.05–0.21)	1	0.03 (0.01–0.12)	0.734
Valvular heart disease		8	0	0	2	0.07 (0.03–0.16)	2	0.07 (0.03–0.16)	4	0.13 (0.07–0.26)	0.618
Other/ uncertain^b^		24	3	0.10 (0.05–0.21)	5	0.17 (0.09–0.30)	7	0.23 (0.14–0.38)	9	0.30 (0.19–0.47)	0.605
**Total No. of events in REC**		31	7	0.40 (0.24–0.66)	8	0.45 (0.28–0.73)	6	0.34 (0.20–0.59)	10	0.57 (0.37–0.87)	0.839
CAD		8	0	0	4	0.23 (0.12–0.44)	1	0.06 (0.02–0.20)	3	0.17 (0.08–0.36)	0.648
	MI	4	0	0	1	0.06 (0.02–0.20)	1	0.06 (0.02–0.20)	2	0.11 (0.05–0.28)	0.645
Cardiomyopathies		9	4	0.23 (0.12–0.44)	0	0	0	0	5	0.28 (0.16–0.51)	0.767
	HCM	6	3	0.17 (0.08–0.36)	0	0	0	0	3	0.17 (0.08–0.36)	1.000
	Possible HCM/Fibrosis^a^	1	0	0	0	0	0	0	1	0.06 (0.02–0.20)	1.000
	ARVC	1	1	0.06 (0.02–0.20)	0	0	0	0	0	0	0.716
	DCM	1	0	0	0	0	0	0	1	0.06 (0.02–0.20)	1.000
Morphological normal heart		4	1	0.06 (0.02–0.20)	1	0.06 (0.02–0.20)	2	0.11 (0.05–0.28)	0	0	0.684
Pulmonary embolism		1	0	0	0	0	0	0	1	0.06 (0.02–0.20)	1.000
Aortic dissection		4	1	0.06 (0.02–0.20)	1	0.06 (0.02–0.20)	2	0.11 (0.05–0.28)	0	0	0.725
Valvular heart disease		3	1	0.06 (0.02–0.20)	1	0.06 (0.02–0.20)	1	0.06 (0.02–0.20)	0	0	0.761
Other/ uncertain^b^		2	0	0	1	0.06 (0.02–0.20)	0	0	1	0.06 (0.02–0.20)	0.804
**Total No. of events in COMP**		21	4	0.91 (0.47–1.76)	9	2.04 (1.31–3.19)	5	1.13 (0.63–2.06)	3	0.68 (0.32–1.46)	0.658
CAD		8	1	0.23 (0.07–0.80)	4	0.91 (0.47–1.76)	2	0.45 (0.19–1.14)	1	0.23 (0.07–0.80)	0.866
	MI	3	1	0.23 (0.07–0.80)	1	0.23 (0.07–0.80)	1	0.23 (0.07–0.80)	0	0	0.732
Cardiomyopathies		5	1	0.23 (0.07–0.80)	2	0.45 (0.19–1.14)	1	0.23 (0.07–0.80)	1	0.23 (0.07–0.80)	0.873
	HCM	2	0	0	0	0	1	0.23 (0.07–0.80)	1	0.23 (0.07–0.80)	0.357
	ARVC	1	0	0	1	0.23 (0.07–0.80)	0	0	0	0	0.905
	DCM	2	1	0.23 (0.07–0.80)	1	0.23 (0.07–0.80)	0	0	0	0	0.304
Myocarditis		1	0	0	0	0	0	0	1	0.23 (0.07–0.80)	0.423
Valvular heart disease		2	1	0.23 (0.07–0.80)	1	0.23 (0.07–0.80)	0	0	0	0	0.304
Other/ uncertain^b^		5	1	0.23 (0.07–0.80)	2	0.45 (0.19–1.14)	2	0.45 (0.19–1.14)	0	0	0.790

^a^ Includes cases of SCD with left ventricular hypertrophy and fibrosis but no myocardial disarray on histologic examination.

^b^ Includes commotio cordis; congenital heart disease; coronary artery anomalies; fibrous dysplasia of the coronary arteries; other forms of cardiomyopathies, systematic diseases; WPW syndrome and uncertain cases (4 cases).

^c^ Time trends were calculated by Poisson log-linear regression analysis.

No case of myocarditis was recorded in REC. There were no deaths with pulmonary embolism, aortic dissection or morphological normal heart on autopsy in COMP.

ARVC, arrhythmogenic right ventricular cardiomyopathy; CAD, coronary artery disease; CI, confidence interval; CMP, cardiomyopathies; COMP, SCD related to competitions; DCM, dilated cardiomyopathy; HCM, hypertrophic cardiomyopathy; MI, myocardial infarction; NONE, SCD not related to physical activities; SCD associated with physical activities other than competitions; SCD, sudden cardiac death

## Discussion

### Incidences and causes of death

Our study revealed an overall low incidence of SCD associated with competitive sports in the young population of Switzerland. The identified incidence rates are comparable to the Italian data in late screening period, and to a study conducted in the US [16, 17]. We observed predominance of male victims in all groups, consistent with other reports [16, 18–21].

Although mortality from cardiovascular disease, and particularly, CAD is very low in Switzerland [22], premature CAD was the most common cause of SCD in all categories. Similar results were observed in the French speaking region of Switzerland [21]. All three professional athletes identified amongst the victims in COMP included in the study succumbed to death due to acute MI. It is worth mentioning that more than 50% of SCDs attributed to CAD in all three categories occurred in the absence of acute MI. It is known that during intensive exercise CAD can cause demand ischemia and cardiac arrest in the absence of acute MI [23], however, this effect is not expected in non-athletic settings (NONE). It is possible that some of the SCDs attributed to CAD in our NONE category were misclassified and these individuals had genetic predisposition to life-threatening arrhythmias, however, these victims had an average age of 35±3.2 years (37±2 years in REC (n = 4); and 35±3.5 years in COMP (n = 5)), therefore channelopathies were less likely responsible for these deaths. It is also known that an unstable plaque that is occluding only 30% to 50% is susceptible to transient coronary artery spasm as an arrhythmia trigger [24].

The two previously mentioned seminal studies from Italy and the USA reported different distributions of underlying causes of SCD in young athletes. Corrado and colleagues have reported a predominant causative role of ARVC in SCD in athletes (25.4%) and a marked decline in cause-specific incidence after implementation of nationwide mandatory ECG-based PPS [16]. In contrast, Maron et al. have found SCD in college athletes to be mainly due to HCM (36%), followed by coronary artery anomalies (17%) [17]. Interestingly, in the same study, HCM was observed 8 times more frequently than ARVC (251 vs. 30 cases). We observed a high number of deaths caused by premature CAD and a low number of cases with ARVC and HCM in our cohort, which is in line with reports from Denmark, Germany, Norway and Ireland [19, 20, 25, 26]. The differences between the aforementioned studies support the theory that regional differences in population characteristics play a central role in the epidemiology of SCD, particularly in athletes [8]. Our study further strengthens the evidence of regional differences in underlying causes of SCD.

### Trends of cardiovascular mortality and pre-participation screening

There is ample evidence of the effectiveness of ECG-based PPS in detecting certain inheritable cardiac conditions. In the Veneto region of Italy, mandatory PPS including history, clinical examination and resting 12-lead ECG substantially decreased the incidence of SCD in young athletes from 4.19 to 0.87 cases per 100,000 person-years. The greatest decline was observed in incidence rates of SCD due to ARVC–the most common cause of SCD in athletes in Veneto, while the decline of SCD related to CAD or other conditions was not significant [16].

In our cohort, the average yearly incidence of COMP due to cardiomyopathies is similar to the rates observed in Veneto over the late screening period (0.28 vs 0.15/100,000 athlete person-years) [16]. We did not find a time trend in yearly incidence of SCD related to cardiomyopathies in COMP after initiation of screening in athletes engaged in high-risk sports, as one might expect. Annual absolute numbers of these cases were very low, which makes the detection of a time trend more difficult.

There are at least three explanations to the overall low incidence of cardiomyopathies and high incidence of CAD in our sample, that are particularly applicable to COMP. Firstly, since the beginning of our data collection nearly coincides with the implementation of ECG-based PPS, the screening may have resulted in the exclusion of individuals with ECG-detectable cardiomyopathies from competitions. Further, all military recruits in Switzerland undergo ECG-based screening, and individuals with disorders detectable on routine ECG may have been informed about potential risks related to the engagement in competitive sports. Alternatively, it might be that the population examined in the present study has lower prevalence of cardiomyopathies and higher rate of CAD.

It appears that PPS including ECG may prevent certain fatal cardiovascular events by detecting underlying inherited/genetic conditions (as ARVC in Veneto), but additional methods are needed to effectively identify candidates for SCD due to silent CAD. In these terms, screening for cardiovascular risk factors such as hypertension, dyslipidemia, and family history of premature CAD may be indicated to decrease mortality from CAD. The importance of detection of CAD as a potential cause of SCD in athletes should be more emphasized in regions where it is the leading cause of fatal cardiovascular events in athletes (e.g., Switzerland, Norway, Germany, Denmark, Ireland) [19, 20, 25, 26].

### Limitations of the study

This paper should be viewed in light of several limitations. The autopsy rate was low (47.5%) due to autopsy being optional, which prevented us from generalizing our findings to all SCD victims. In Switzerland, postmortem diagnosis is made by experienced forensic pathologists, who are not necessarily specialized in cardiovascular pathology. Nevertheless, applying the high diagnostic yield of histological examination and toxicological screening assures high diagnostic accuracy. A study in college athletes in the US suggested that a multidisciplinary expert-panel for comprehensive evaluation of pathological findings may improve the classification of underlying causes, particularly of cardiomyopathies [27]. However, post-mortem diagnosis of CAD–the most frequent underlying pathology in all categories in our study, is simple, unlike the detection of non-obvious ARVC, HCM or myocarditis, which may require detailed microscopic examination. Moreover, acute coronary occlusion due to thrombosis as the cause of acute MI is a diagnosis not-to-miss and a certain cause of SCD [28].

It is possible that the NONE category included athletes who died in circumstances unassociated with exercise [17], during routine daily activities or while asleep, when increased vagal tone favors slower heart rate, such as in Brugada syndrome or Long QT syndrome type 3 [29, 30]. However, this does not affect the results of this study, as we examined SCDs related to competitions, rather than investigating SCDs in competitive athletes, therefore, the rates and incidences observed should not be mistakenly assigned to athletes or non-athletes.

We have no explanation for the SCDs with morphological normal heart on autopsy. These deaths could be related to inherited cardiac channelopathies but postmortem genetic testing is not routinely perform in forensic pathology. The denominators for sports-participation were based on survey results. However, our incidences are comparable to those of other studies [16]. Given the higher proportion of witnessed cases in COMP and REC, compared to NONE, the rates of sports-related SCD more reliably reflect the real incidences, while the rate in NONE may be slightly underestimated due to lower witness (and autopsy) rate. Considering also the adjustment for autopsy rate, the reported incidences for REC and COMP are at the upper margin of the expected rates and are certainly not underestimated.

## Conclusions

The incidence of SCD in the German-speaking part of Switzerland was low. CAD was the predominant cause of SCD in sports-related and unrelated categories. Screening for cardiovascular risk factors such as tobacco smoking, hypertension, dyslipidaemia, and family history of premature CAD in addition to the current PPS recommendations might be indicated to improve detection of silent CAD and further decrease the incidence of SCD.

## Supporting information

S1 Dataset Characteristics of sudden cardiac death victims. (XLSX)

S1 Appendix Supporting information to the dataset. (DOCX)
