# Supplementary material for: Sports-related sudden cardiac deaths in the young population of Switzerland
Source: PLoS One. 2017 Mar 28;12(3):e0174434. doi: 10.1371/journal.pone.0174434 (PMC5370100; doi:10.1371/journal.pone.0174434)
Supplement: S1 Appendix — (DOCX) [file pone.0174434.s002.docx]

**Supporting information to the dataset**

In the dataset, diagnosis is classified as:

0=morphological normal heart

1=hypertrophic cardiomyopathy

2=possible hypertrophic cardiomyopathy/fibrosis

3=arrhythmogenic right ventricular cardiomyopathy

4=dilated cardiomyopathy

5=coronary artery disease - without myocardial infarction

6= coronary artery disease - myocardial infarction

7=coronary anomaly

8=fibrous muscular dysplasia of coronary artery

9=myocarditis

10=mitral valve prolapse

11=valve stenosis

12=aortic dissection

13=pulmonary embolism

14=systematic disease

15=other cardiomyopathy

16 congenital heart disease

17=WPW syndrome*

18=commotio cordis

19=other/unclear.

All the other descriptions are provided in the database.

For the calculation of incidences, we used the following population data:

|  | Average number of inhabitants aged 10-39 years in the examined region between 1999-2010[1] |
| --- | --- |
| **Total** | 2 112 038 |
| Male | 1 072 209 |
| Female | 1 039 829 |
| **Population engaged in sports (73%)**[2] | 1 541 788 |
| Male | 782 713 |
| Female | 759 075 |
| ***Competitive (20%)*** | 308 358 |
| ***Recreational (80%)*** | 1 233 430 |

1. Swiss Federal Office of Statistics: Available at: <http://www.bfs.admin.ch/bfs/portal/en/index/infothek/erhebungen__quellen/blank/blank/statpop/01.html>. Accessed February 21, 2015.

2. Swiss Federal Office of Sports. Available at: <http://www.baspo.admin.ch/internet/baspo/de/home/dokumentation.parsys.0001101.downloadList.17485.DownloadFile.tmp/basposportschweizde.pdf>. Accessed May 15, 2011.
